# Continued use of Warfarin in lower dose has safe maternal and neonatal outcomes in pregnant women with Prosthetic Heart Valves

**DOI:** 10.12669/pjms.37.4.3924

**Published:** 2021

**Authors:** Shafaq Nadeem, Shabaz Ahmad Khilji, Faisal Ali, Anjum Jalal

**Affiliations:** 1 Shafaq Nadeem, FCPS. Consultant of Gynecology & Obstetrics The Clinic for Women with Cardiac Diseases, Department of Cardiac Surgery, Faisalabad Institute of Cardiology, Faisalabad, Pakistan; 2 Shahbaz Ahmad Khilji, FCPS. Associate Professor Department of Cardiac Surgery, Faisalabad Institute of Cardiology, Faisalabad, Pakistan; 3 Faisal Ali, Dip Card. Consultant Cardiologist, Department of Cardiology, Faisalabad Institute of Cardiology, Faisalabad, Pakistan; 4 Anjum Jalal, FRCS-CTh. Professor of Cardiac Surgery, Executive Director, Faisalabad Institute of Cardiology, Faisalabad, Pakistan

**Keywords:** Prosthetic Heart Valve, Anticoagulation, Warfarin, Heparin related Embryopathy, Warfarin in Pregnancy

## Abstract

**Background and Objective:**

There has been concerns regarding the safety of Warfarin in pregnant females due to its teratogenic potential. At the same time warfarin provides best anticoagulation in patients with prosthetic valves. Various dosage regimes have been tried to strike a balance between safety of mother and the avoidance of congenital anomalies in the newborn. This study was conducted to observe the effect of Warfarin in pregnant mothers taking different doses of warfarin, and their neonatal outcome, in our outdoor patients.

**Methods:**

This is a cross sectional observational study conducted at the Faisalabad Institute of Cardiology. The pregnant mothers taking warfarin for prosthetic valve replacement who presented to our specialized clinic between November 2016 to April 2017 were included in the study. These included a total of 75 females between the age of 20-35 years. To compare the dose related effect of warfarin, two groups of the patients were formed. One group comprised of patients taking warfarin ≤5mg while the other group consisted of those who were taking >5mg of warfarin daily. These patients were followed till their delivery. The information was collected about the maternal and fetal outcomes. The maternal outcomes including mode of delivery/miscarriage, peripartum bleeding and any valve related thromboembolic complications. The fetal outcomes included birth weight, maturity, embryopathy and congenital anomaly in the baby.

**Results:**

Patient’s mean age was 29.25±3.75 years. The mitral valve replacement was present in 60% patients (n=45) while 25.3% patients (n=19) had aortic valve replacement and 14.7% patients (n=11) had double valve replacement. In this group 30 patients (40%) had taken <5 mg warfarin and 45 patients (60%) had received >5 mg warfarin medicine. Miscarriages, cesarean sections, low birth weight and prematurity were more common in patients receiving warfarin >5 mg with p-values 0.005, 0.046, 0.01 and 0.033 respectively. No case of fetal embryopathy was found in both groups.

**Conclusion:**

No case of embryopathy was found in each group which signifies that warfarin in lower doses is safe anticoagulant in patients with prosthetic valve replacements.

## INTRODUCTION

The valve replacement with mechanical prosthesis provides excellent long term freedom from symptoms as it improves the cardiac dimensions, recovers cardiac functional status, and prolongs overall survival.^1^ However, it necessitates the use of warfarin for long term anticoagulation. In order to maintain the therapeutic levels of warfarin, regular titration of dose is required with periodic testing of prothrombin time. Warfarin has the ability of crossing the placental barrier and might have teratogenic risk during early stages of pregnancy. It is thought that the greatest risk of warfarin embryopathy occurs at eight weeks of pregnancy.^2^ In addition to fetal complications, warfarin can cause serious bleeding at the time of delivery. Contrarily, cessation of warfarin or substitution of warfarin with other anticoagulants can result in prosthetic valve thrombosis which is potentially fatal condition for the mother. Very scarce international and local data is available on dose related teratogenicity of warfarin. There are no controlled clinical trials to provide guidelines for optimal anti-thrombotic therapy. Currently, three regimens of anticoagulation are followed across various different countries globally.^3^ These include:

1. Warfarin sodium throughout pregnancy with unfractionated heparin sodium near term,
2. Substitution of warfarin with unfractionated heparin between 6 and 12 weeks of gestation and near term, and
3. Unfractionated heparin throughout pregnancy.

Evidence supporting the use of these regimens is poor and is derived from case reports, case series, small cohort studies, and questionnaires. Considering these difficulties observed in under developed countries, attempts have been made to simplify the management protocols in this subset of patients. There is some evidence that self monitoring of INR and keeping a lower target INR with relatively lower dose of warfarin results in much lower bleeding complications without increasing the risk of valve thrombosis in general population.^4^ There is also some evidence that keeping a slightly lower INR within a range of 2.0 to 2.5 can avoid many complications in Pakistani patients.^5^

The impact of women with prosthetic valves on maternal mortality would increase as with the availability of cardiac surgery services this segment of population is growing in Pakistan. It has been reported that in UK the incidence of women with prosthetic valves is 3.7 per 100000 maternities and there is significantly high rate of maternal death, and serious maternal and fetal morbidity amongst those who took low molecular weight heparin (LMWH).^6^ Our study was aimed at finding the effect of different doses of warfarin on maternal and neonatal outcomes, in our outdoor patients. The results of this study would be helpful to highlight the simplicity and safety of using low dose warfarin throughout the pregnancy.

## METHODS

This study was conducted on the patients attended at the Faisalabad Institute of Cardiology (FIC) in cardiac surgery OPD in collaboration with consultant cardiac surgeon, consultant obstetrician and consultant cardiologist for duration of six months starting from November 2016 to April 2017. It was a cross sectional observational study of 75 women of age 20-35 years who had prosthetic valve replacement and remained on warfarin for anticoagulant throughout pregnancy. The INR was kept within the therapeutic range (2.5-3.5). The exclusion criteria included previous history of recurrent miscarriages, previous births of babies with congenital anomalies, existence of chronic debilitating ailments in mother like, diabetes, hypertension, tuberculosis, malnutrition, and pulmonary hypertension.

The ethical committee of the hospital approved the study vide its Letter No# 01-2020/DME/FIC/FSD, Dated 11.07.2020. All patients received detailed counseling before signing their informed consent to be part of the study. For calculation of sample size a prematurity prevalence of 14.3% as reported previous was taken as reference.^4^ Using WHO calculator, with prevalence rate of 14.3%, confidence level of 95% and precision of 8%, the sample size came out to be 74 patients. Non-probability, consecutive sampling technique was implied for enrolling individuals. After enrolling all the patients the dose which resulted in NR level in therapeutic range (2.5-3.5) was noted. The patients were then classified into Low dose and High dose groups. Those patients who were taking 5mg or less of warfarin were put into Low dose group and those who were taking more than 5mg of warfarin daily were classified into High dose group. The information was collected about maternal and fetal outcomes. The maternal outcomes including mode of delivery/miscarriage, peripartum bleeding and any valve related thromboembolic complications. The fetal outcomes included birth weight, maturity, embryopathy and congenital anomaly in the baby. The SPSS version 17 was used to analyze data. Mean as well as standard deviation was obtained for all quantitative variables like age of the patient, gestational age and parity etc. Frequencies as well as percentages were calculated for categoric variables. To compare the effect of dose of warfarin on maternal and neonatal outcomes Chi-square test was used. The P-value of ≤0.05 was taken as significant.

## RESULTS

The characteristics of the study population is shown in Table-I. The mean age of the patients was 29.25 (standard deviation ± 3.75) years. The mean gestational age of the patients was 31.79 (± 9.85) weeks. The mean parity was 2.2 (± 0.81). The mitral valve replacement was present in 45 patients (60%). The aortic valve replacement was found in 19 (25.3%) patients, while 11 (14.7%) patients were found to have double valve replacement. It was observed that 30 (40%) patients were taking <5 mg warfarin while 45 (60%) patients were taking >5 mg warfarin daily. The maternal outcome of warfarin is shown in Table-II. Out of 75 patients, 21 (28%) patients had miscarriage. Cesarean section were conducted on 35 (64.8%) patients because of obstetric or medical reasons and 19 (35.2%) patients had normal vaginal delivery. Miscarriage and Cesarean section was significantly more common among the patients taking higher dose of warfarin (P = 0.005 and 0.046 respectively). The neonatal outcome of warfarin is shown in Table-III. Out of 75 patients, 19 (35.2%) had low birth weight babies, 15 (27.8%) had premature babies and no case of embryopathy was found. There is statistically significant difference regarding low birth weight and prematurity between two groups with p-values 0.01 and 0.033 respectively.

**Table-I:** Characteristics of study population.

Demographic	Warfarin < 5mg n=30	Warfarin >5mg n=45	All
Age (Years)	29.03±3.03	29.4±4.18	29.25±3.75
Gestational Age (Weeks)	36.57±4.64	28.6±11.1	31.79±9.85
Parity	2.17±0.79	2.22±0.82	2.2±0.81
***Type of Valve Surgery***			
Mitral	20 (66.7%)	25 (55.6%)	45 (60%)
Aortic	5 (16.7%)	14 (31.1%)	19 (25.3%)
Double	5 (16.7%)	6 (13.3%)	11 (14.7%)

**Table-II:** Maternal outcome.

Outcome	Warfarin < 5mg n=30	Warfarin >5mg n=45	Total	P
Miscarriage	3 (10%)	18 (40%)	21 (28%)	0.005
***Mode of Delivery***				
Vaginal	13 (48.1%)	6 (22.2%)	19 (35.2%)	0.046
Cesarean Section	14 (51.9%)	21 (77.8%)	35 (64.8%)	

**Table-III:** Neonatal outcome.

Outcome	Warfarin < 5mg n=30	Warfarin >5mg n=45	Total	P
Low Birth Weight	5 (18.5%)	14 (51.9%)	19 (35.2%)	0.01
Prematurity	4 (14.8%)	11 (40.7%)	15 (27.8%)	0.033
Embropathy	Nil	Nil	Nil	

## DISCUSSION

The aim of “live mothers for live neonates” is the fundamental desire of society around which most of the policy making is centered in the modern healthcare. Prosthetic valve replacement in the female patients of child bearing age, has created a great dilemma in the management of anticoagulation during pregnancy. The potential of teratogenicity of warfarin, is a sensitive issue. The role of warfarin as an anti-coagulant in pregnancy has always been a topic of conflict and debate. It is observed that both patients and obstetricians have confusing views and fears about the use of warfarin during pregnancy particularly in its first trimester. It is also observed in our practice that many mothers suffer from valve thrombosis resulting from self-initiated cessation of warfarin to avoid fetal abnormalities. It is established that warfarin is more effective than any other form of anticoagulation including unfractionated heparin in pregnant women with mechanical valves. Unluckily, warfarin administration has been ascribed to a greater prevalence of fetal miscarriages, low birth weight babies and prematurity. Attempts have been made to study the safety of low dose warfarin during pregnancy.

Ayad et al observed a total of 60 patients which included mitral valve replacement in 20 patients, aortic valve replacement among 22 patients and that of double valve replacement in 18 patients. Most of these patients (n=47) underwent cesarean deliveries, while spontaneous normal vaginal deliveries were seen in 24 patients.^7^ They observed miscarriage rate was 6.1%, in patients taking warfarin dose ≤ 5 mg while miscarriage rate of 41.5% was observed in patients taking warfarin dose > 5 mg. There was no case of warfarin related embryopathy in both groups. In another study, Tounsi et al demonstrated undesirable consequences were observed more commonly in patients who took warfarin in dose of 5 mg daily or more.^8^ Samiei et al observed high rate of miscarriage (34.9%), cesarean section (65.4%) and prematurity (7%) in patients taking warfarin dose >5mg per day.^9^ They recommended that warfarin is a safe anticoagulant during pregnancy in lower doses. Mazibuko et al observed the miscarriage rate of 20% and embryopathy in 7% cases in women who took >5 mg warfarin daily during their pregnancy.^10^ Vitale et al observed that a close relationship was present between warfarin dose and fetal complications among pregnant women having mechanical prosthetic heart valve replacement.^11^ They included 58 pregnancies in their study and showed that the dosage of >5 mg warfarin daily was associated with majority of fetal complications. Out of thirty three women having <5 mg warfarin during pregnancy, only one case (3.03%) of premature baby and four cases (12.12%) of miscarriage were observed while in women taking > 5 mg of warfarin embryopathy were noted in two out of 25 patients (8%). Sadler et al demonstrated that seven miscarriages out of 11 women treated with >5 mg daily warfarin compared to 5 miscarriages out of 11 women treated with <5 mg daily warfarin.^12^ McLintock et al observed improved maternal safety with warfarin as predominant oral anticoagulant therapy but had relatively poor fetal outcomes.^13^ Nassar et al showed miscarriage rate of 23.7% in women taking warfarin throughout pregnancy.^14^ In a review article by Steinberg et al, concluded that warfarin based anticoagulation was associated with better maternal outcomes, whereas the use of LMWH during pregnancy was associated with better fetal outcomes. It was further reassuring that warfarin in daily dose of ≤5 mg was as safe as LMWH in terms of fetal outcomes.^15^

The American Heart Association has simplified its guidelines regarding anticoagulation during pregnancy in the form of algorithm based on the best available evidence (Fig.1).^16^ Our experience reported in this study also highlights that a vast majority of pregnant ladies in Pakistan can be safely continued on lose dose of warfarin throughout pregnancy. We believe this information needs to be disseminated through the medical community as well as the young female patients at the time of their valve surgery to avoid the unnecessary incidences of potentially fatal prosthetic valve thrombosis.

**Figure:**
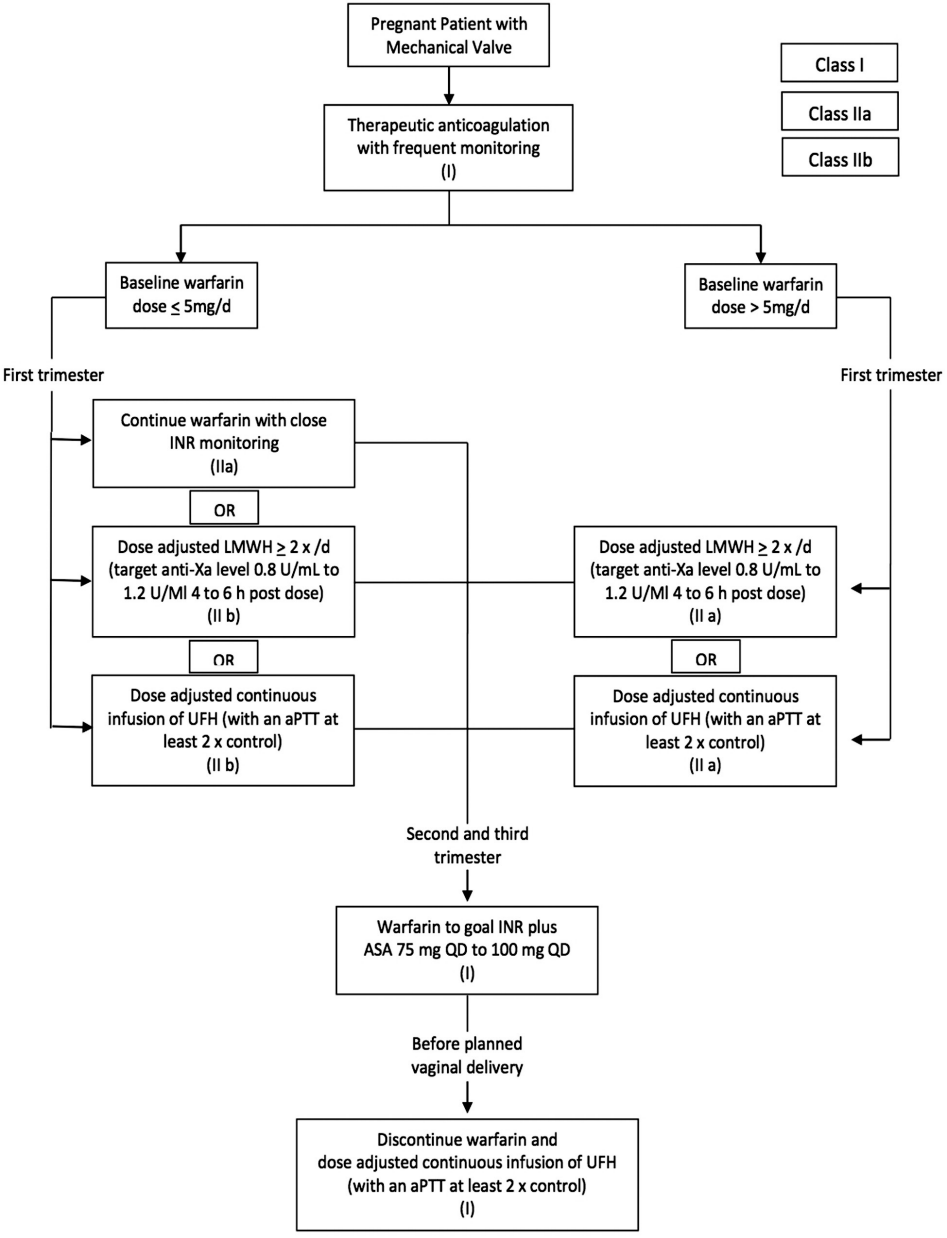
Fig.1

### Limitation of the study

It is a cross sectional study conducted at one highly specialized center. The total number of patients in the sample is also very small. The vast majority of the patients in the study were from the surrounding of Faisalabad city which is relatively better developed administrative division of Punjab province having better access to the healthcare facilities. It may not reflect the picture from poorly developed areas where the complications rates ascribed to cessation of warfarin may be much higher.

## CONCLUSION

We conclude that the warfarin in lower doses should be considered safe anti-coagulation agent throughout pregnancy after prosthetic valve replacement. We believe that the risk of warfarin induced embryopathy is over emphasized and is resulting in unnecessary maternal mortality and morbidity due valve thrombosis and this trend must be stopped in our population.

### Authors’ Contribution:

**SN & SAK:** Conceived, designed, collected data, prepared first draft of manuscript and are responsible for integrity of the study.

**FA:** Helped in data collection and echocardiographic evaluation during follow-up.

**AJ:** Supervised the study, prepared revised manuscript and gave final approval of manuscript.
